# Supplementary material for: Cullin-associated and neddylation-dissociated protein 1 (CAND1) alleviates NAFLD by reducing ubiquitinated degradation of ACAA2
Source: Nat Commun. 2023 Aug 1;14:4620. doi: 10.1038/s41467-023-40327-5 (PMC10394019; doi:10.1038/s41467-023-40327-5)
Supplement: Supplementary file 1 — Supplementary Information [file 41467_2023_40327_MOESM1_ESM.pdf]

Supplementary Information

Cullin-associated and neddylation-dissociated protein 1 (CAND1) alleviates NAFLD by reducing ubiquitinated degradation of ACAA2

Xiang Huang [1, #], Xin Liu [2, #], Xingda Li [3, #], Yang Zhang [1, #], Jianjun Gao [4], Ying Yang [1], Yuan Jiang [5], Haiyu Gao [1], Chongsong Sun [1], Lina Xuan[1], Lexin Zhao [1], Jiahui Song [1], Hairong Bao [1], Zhiwen Zhou [1], Shangxuan Li [1], Xiaofang Zhang [1], Yanjie Lu [1, *], Xiangyu Zhong [4, *], Baofeng Yang[1, 6, 7, *] Zhenwei Pan [1, 6, 8, *]

1. Department of Pharmacology (National Key Laboratory of Frigid Zone Cardiovascular Disease, Key Laboratory of Cardiovascular Research. Ministry of Education), College of Pharmacy, Harbin Medical University, Harbin, Heilongjiang 150086, P. R. China.

2. The Department of Histology and Embryology, Harbin Medical University, Harbin 150086, China.

3. Department of Pharmacy at the Second Affiliated Hospital, and Department of Pharmacology at College of Pharmacy (The Key Laboratory of Cardiovascular Research, Ministry of Education), Harbin Medical University, Harbin 150086, China

4. The Department of Hepatopancreatobility, Surgery Second Affiliated Hospital of Harbin Medical University, 150086, China.

5. Medical Research Center, Sun Yat-Sen Memorial Hospital, Sun Yat-Sen University, Guangzhou, 510120, China.

6. Research Unit of Noninfectious Chronic Diseases in Frigid Zone, Chinese Academy of Medical Sciences, 2019 Research Unit 070, Harbin, Heilongjiang 150086, P. R.

China.

7. State Key Laboratory, Harbin Medical University, Harbin 150086, China.

8. Key Laboratory of Cell Transplantation, The First Affiliated Hospital, Harbin Medical University, Harbin 150086, China.

[#] Xiang Huang, Xin Liu, Xingda Li and Yang Zhang contributed equally to this study;

Corresponding author: Prof. Zhenwei Pan. panzw@ems.hrbmu.edu.cn.

Department of Pharmacology, College of Pharmacy, Harbin Medical University, Baojian Road 157, Harbin, Heilongjiang 150081, P. R. China. Fax: 86 451 86675769 or 86667511 Tel: 86 451 86671354

Prof. Baofeng Yang. yangbf@ems.hrbmu.edu.cn. Department of Pharmacology, College of Pharmacy, Harbin Medical University, Baojian Road 157, Harbin, Heilongjiang 150081, P. R. China. Fax: 86 451 86675769 or 86667511 Tel: 86 451 86671354

Prof. Xiangyu Zhong. zhongxiangyu@hrbmu.edu.cn. Department of Hepatopancreatobiliary Surgery, Second Affiliated Hospital of Harbin Medical University, Harbin, China.

Prof. Yanjie Lu. yjlu@hrbmu.edu.cn. Department of Pharmacology, College of Pharmacy, Harbin Medical University, Baojian Road 157, Harbin, Heilongjiang 150081, P. R. China. Fax: 86 451 86675769 or 86667511 Tel: 86 451 86671354

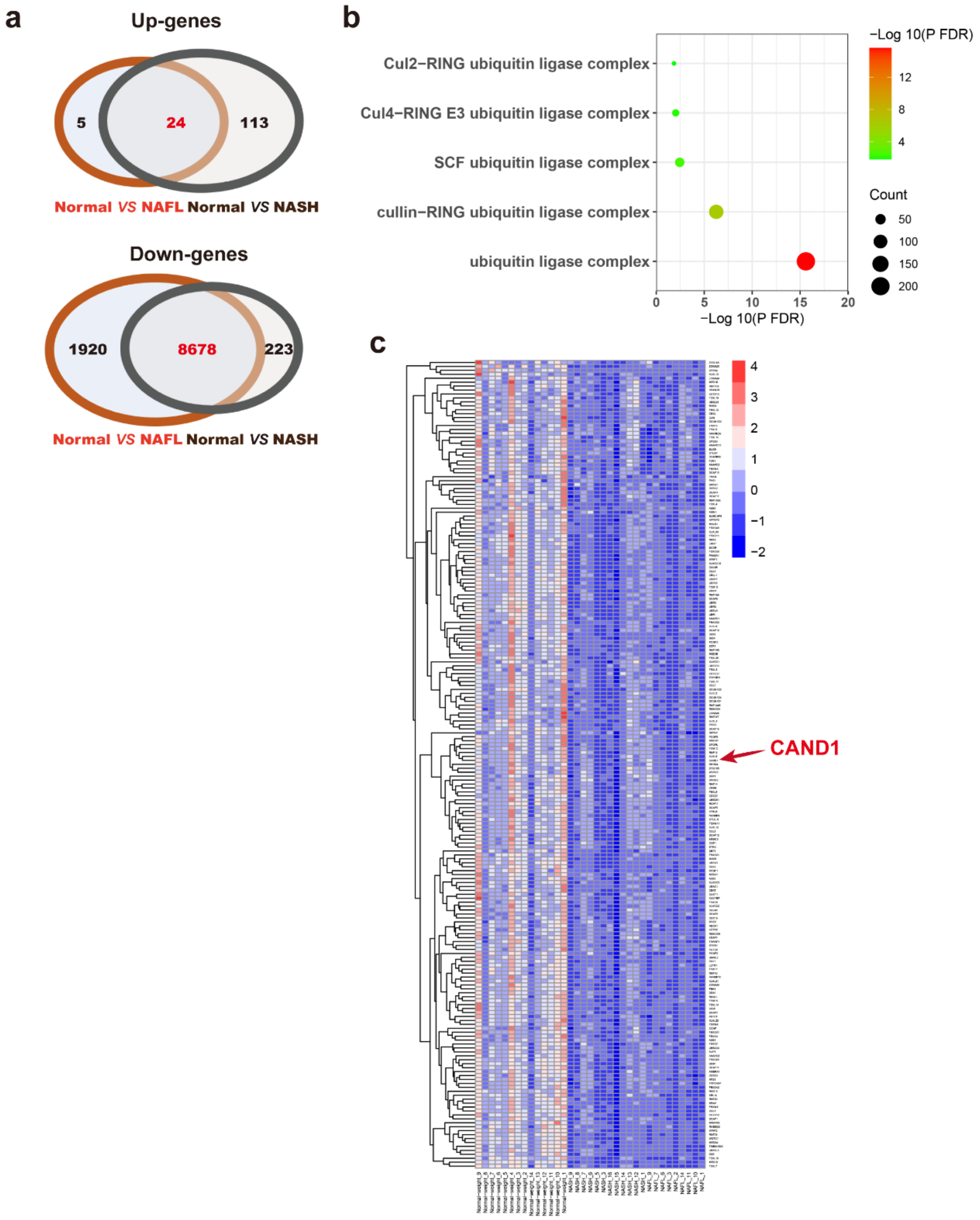

Supplementary Figure 1. Deregulated genes in male patients with NAFLD. (a) Venn diagram highlighting significantly upregulated and downregulated genes in NAFL and NASH patients compared to Normal volunteers. (b) Enriched GO term process associated with protein ubiquitination from gene enrichment analysis on overlapping genes. (c) Heap map depicting relative expression of genes involved with

protein ubiquitination. Source data are provided as a Source data file.

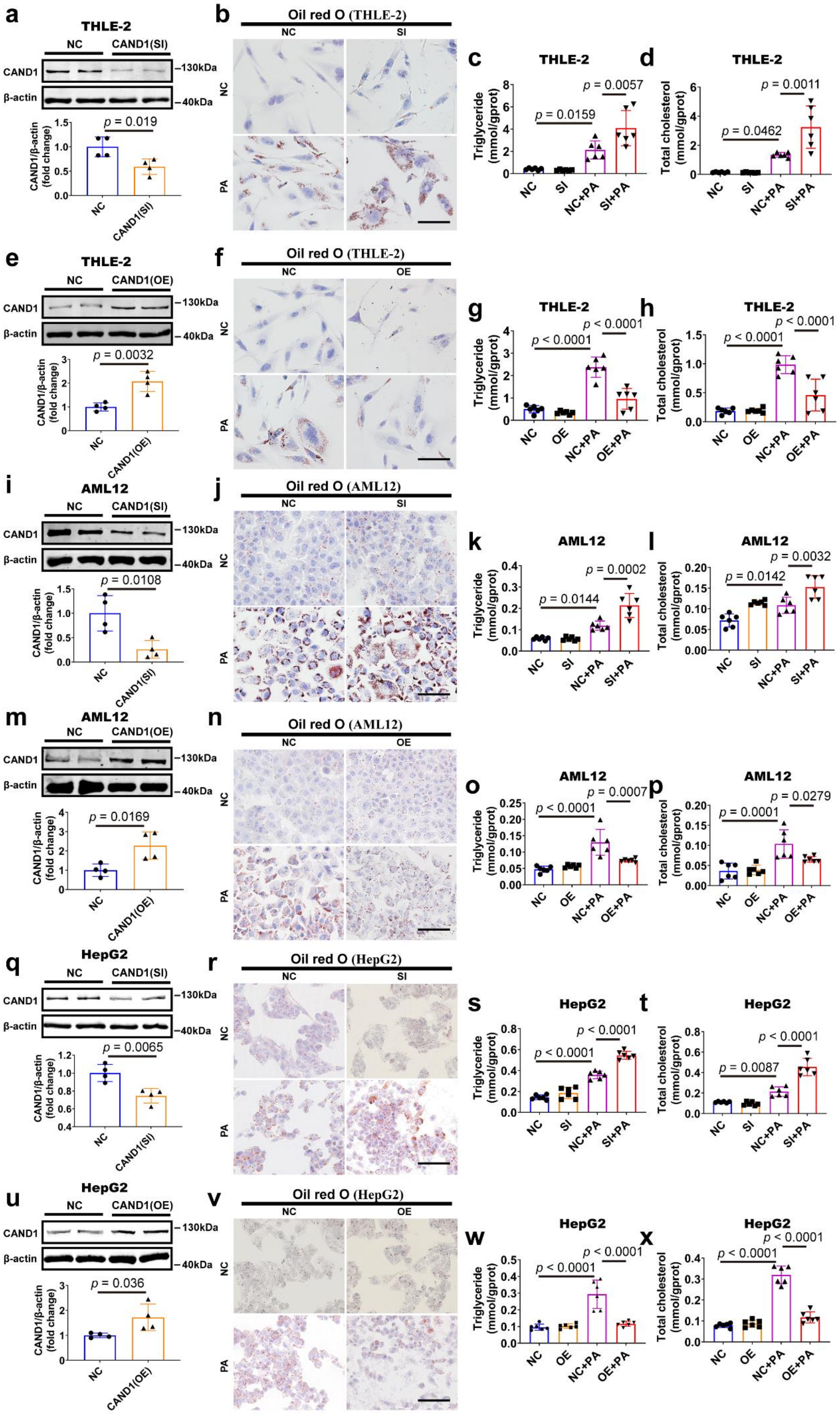
a
THLE-2
NC
CAND1(SI)
CAND1
β-actin
130kDa
40kDa
CAND1/β-actin (fold change)
p = 0.019
b
Oil red O (THLE-2)
NC
SI
PA
c
THLE-2
Triglyceride (mmol/gprot)
p = 0.0159
p = 0.0057
NC
SI
NC+PA
SI+PA
d
THLE-2
Total cholesterol (mmol/gprot)
p = 0.0462
p = 0.0011
e
THLE-2
CAND1(OE)
p = 0.0032
f
Oil red O (THLE-2)
OE
g
THLE-2
p < 0.0001
OE+PA
h
THLE-2
i
AML12
p = 0.0108
j
Oil red O (AML12)
k
AML12
p = 0.0144
p = 0.0002
l
AML12
p = 0.0142
p = 0.0032
m
AML12
p = 0.0169
n
Oil red O (AML12)
o
AML12
p = 0.0007
p
AML12
p = 0.0001
p = 0.0279
q
HepG2
p = 0.0065
r
Oil red O (HepG2)
s
HepG2
t
HepG2
p = 0.0087
u
HepG2
p = 0.036
v
Oil red O (HepG2)
w
HepG2
x
HepG2

Supplementary Figure 2. CAND1 alleviates PA-induced lipid accumulation in cultured THLE-2, AML12 and HepG2 cells. (a) CAND1 knockdown efficiency by Western blotting (n=4). (b) Oil red O staining of PA treated THLE-2 cells after knockdown of CAND1 (n=6). (c, d) Knockdown of CAND1 on TG and TC content of PA treated THLE-2 cells (n=6). (e) Western blotting of CAND1 in THLE-2 cells transfected with CAND1 overexpressing plasmids (n=4). (f) Oil red O staining of PA treated THLE-2 cells after CAND1 overexpression (n=6). (g, h) TG and TC content of PA treated THLE-2 cells after CAND1 overexpression (n=6). (i) CAND1 knockdown efficiency by Western blotting (n=4). (j) Oil red O staining of PA treated AML12 cells after knockdown of CAND1 (n=6). (k, l) Knockdown of CAND1 on TG and TC content of PA treated AML12 cells (n=6). (m) Western blotting of CAND1 in AML12 cells transfected with CAND1 overexpressing plasmids (n=4). (n) Oil red O staining of PA treated AML12 cells after CAND1 overexpression (n=6). (o, p) TG and TC content of PA treated AML12 cells after CAND1 overexpression (n=6). (q) CAND1 knockdown efficiency by Western blotting (n=4). (r) Oil red O staining of PA treated HepG2 cells after knockdown of CAND1 (n=6). (s, t) Knockdown of CAND1 on TG and TC content of PA treated HepG2 cells (n=6). (u) Western blotting of CAND1 in HepG2 cells transfected with CAND1 overexpressing plasmids (n=4). (v) Oil red O staining of PA treated HepG2 cells after CAND1 overexpression (n=6). (w, x) TG and TC content of PA treated HepG2 cells after CAND1 overexpression (n=6). *p* values obtained via two-tailed unpaired Student's *t* tests, one-way ANOVA with Tukey's multiple comparisons test. The data were shown as means ± SD of independent biological replicates. Source

data are provided as a Source data file. TG, Triglyceride; TC, Total cholesterol; NC, negative control. Scale bar=50 μm. Magnification 200×.

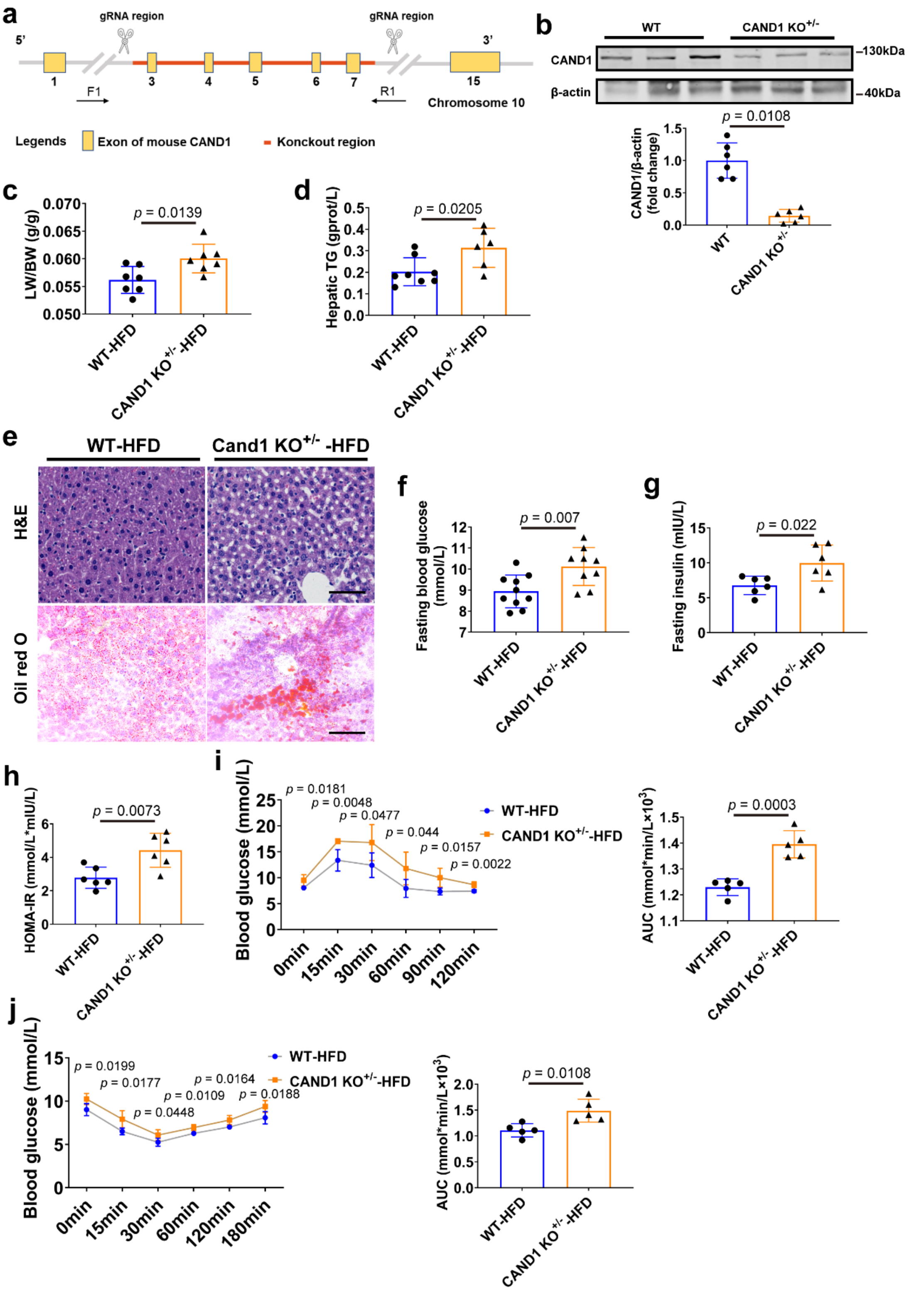

p = 0.0188

Supplementary Figure 3. Conventional CAND1 heterozygous knockout (CAND1 KO$^{+/-}$) aggravates HFD-induced liver steatosis and insulin resistance. (a) Schematic

diagram of CAND1 KO mice using CRISPR/Cas9 system. The deleted region (exons3-7) of CAND1 is indicated in the sequencing results. (b) Western blotting of CAND1 in WT and CAND1 $KO^{+/-}$ mice (n=6). (c) Liver weight to body weight ratio (LW/BW) of CAND1 $KO^{+/-}$ and WT mice after 12 weeks of HFD feeding (n=7). (d) TG content in the indicated groups (WT-HFD, n=8; CAND1 $KO^{+/-}$-HFD, n=6). (e) H&E and Oil red O staining of liver sections of CAND1 $KO^{+/-}$ and WT mice after 12 weeks of HFD treatment (n=6). (f) Fasting blood glucose of WT and CAND1 $KO^{+/-}$ mice after 12 weeks of HFD feeding (WT-HFD, n=10; CAND1 $KO^{+/-}$-HFD, n=9). (g) Fasting insulin levels for WT and CAND1 $KO^{+/-}$ mice after 12 weeks of HFD feeding (n=6). (h) HOMA-IR index for WT and CAND1 $KO^{+/-}$ mice after 12 weeks of HFD feeding (n=6). (i) GTTs of WT and CAND1 $KO^{+/-}$ mice after 12 weeks of HFD feeding (n=5). (j) ITTs of WT and CAND1 $KO^{+/-}$ mice after 12 weeks of HFD feeding (n=5). *p* values obtained two-sided unpaired Student's *t*-test. The data were shown as means ± SD of independent biological replicates. Source data are provided as a Source data file. TG, Triglyceride. Scale bar=50 μm. Magnification 200×.

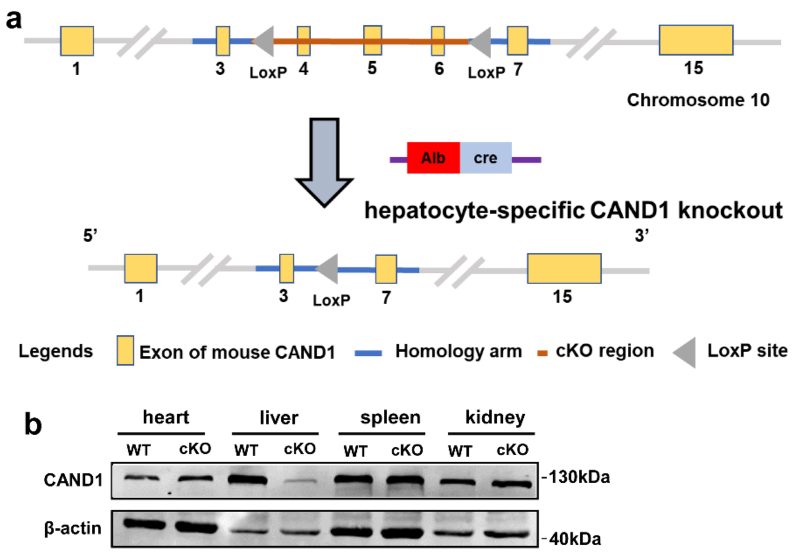

Supplementary Figure 4. Construction of CAND1 hepatocyte-specific knockout (conditional knockout, cKO) mice. (a) A simple schematic diagram for the generation of CAND1 cKO mice. (b) Western blotting of CAND1 in WT and cKO mice (n=4). Source data are provided as a Source data file.

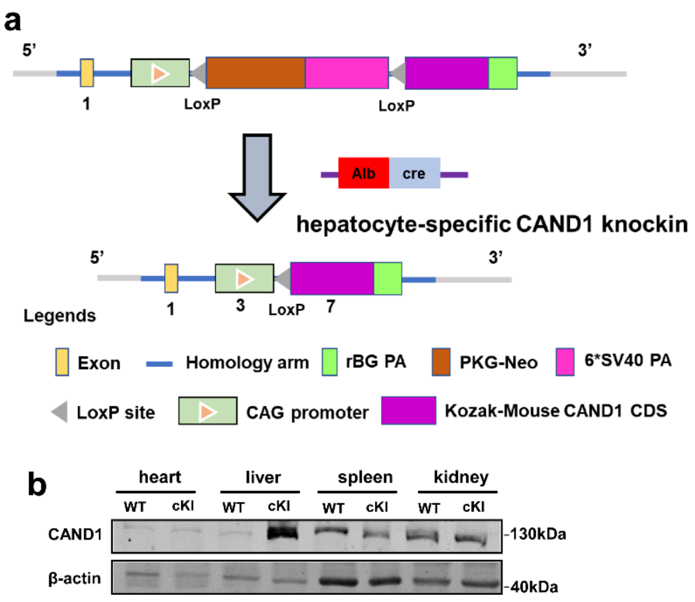

Supplementary Figure 5. The construction of CAND1 hepatocyte-specific knockin (cKI) mice. (a) A simple schematic diagram for the generation of CAND1 cKI mice. (b) Western blotting of CAND1 in WT and cKI mice (n=4). Source data are provided as a Source data file.

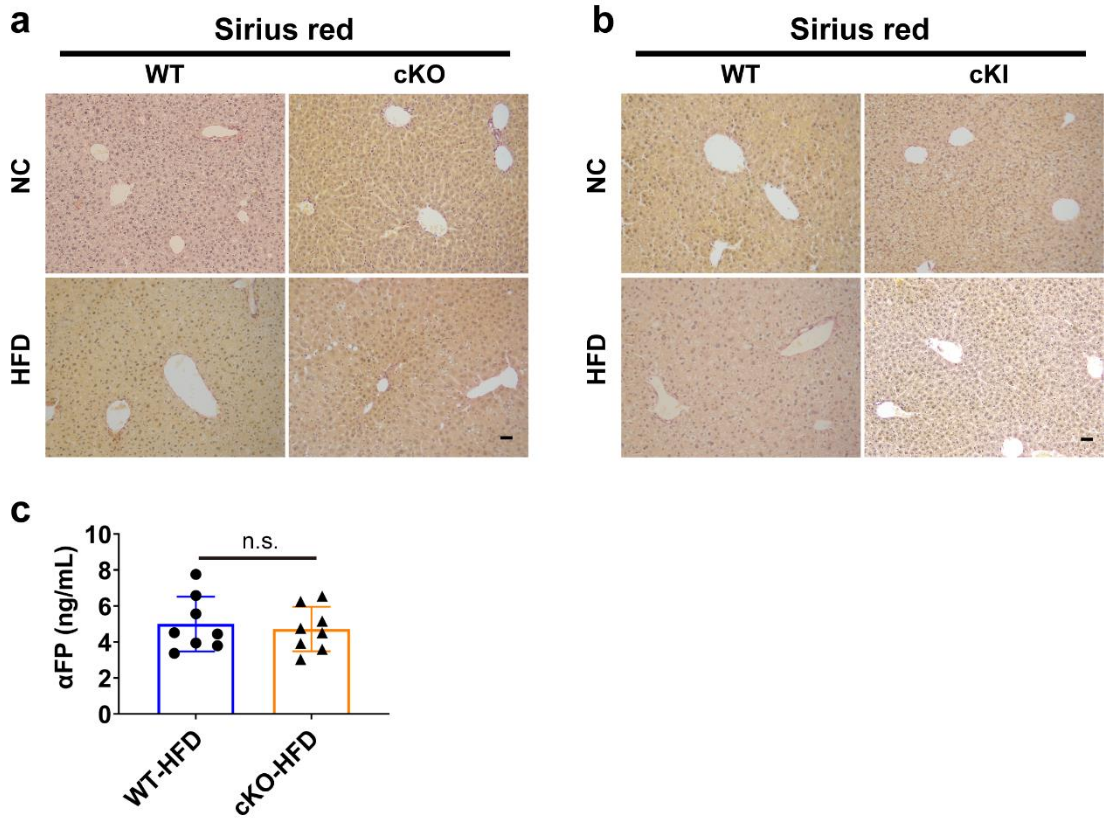

Supplementary Figure 6. CAND1 has no effect on fibrosis in NAFLD male mice. (a) Sirius red staining of liver sections of cKO and WT male mice after 16 weeks of HFD treatment (n=5). (b) Sirius red staining of liver sections of cKI and WT male mice after 16 weeks of HFD treatment (n=5). (c) αFP levels in cKO and WT male mice after 16 weeks of HFD treatment (n=8). *p* values obtained two-sided unpaired Student's *t*-test. The data were shown as means ± SD of independent biological replicates. Source data are provided as a Source data file. n.s., not significant difference. Scale bar=50 μm. Magnification 100×.

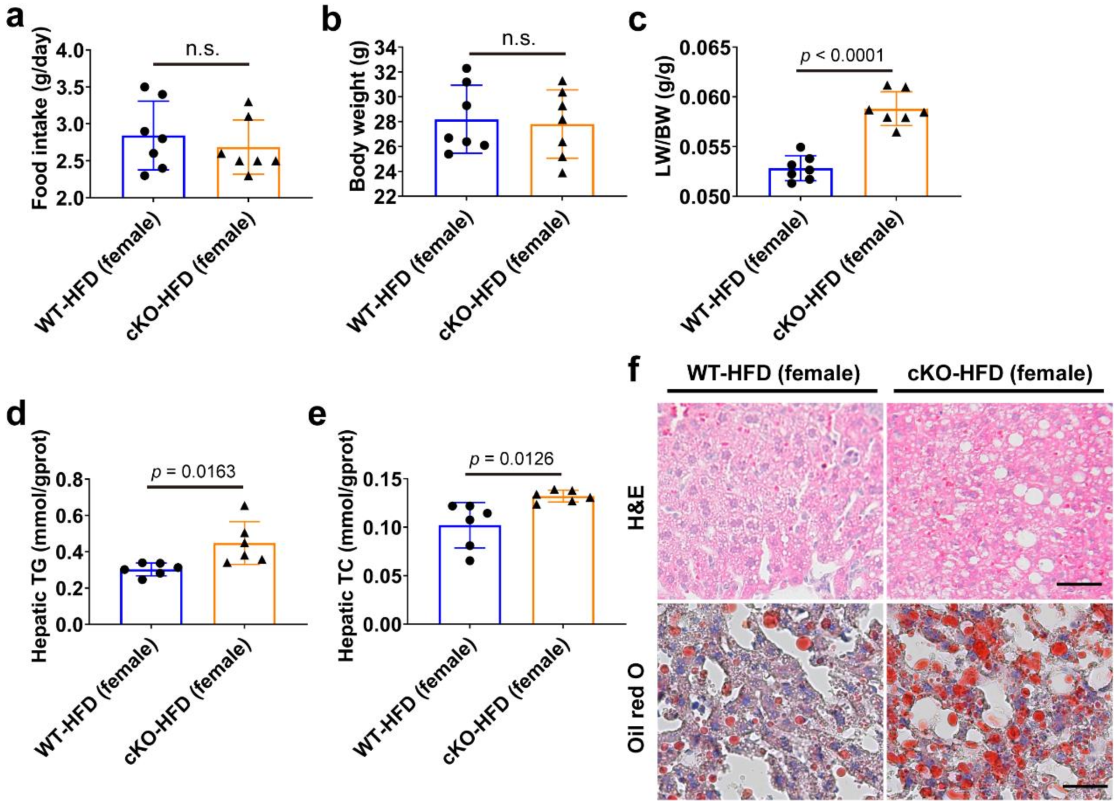

Supplementary Figure 7. Hepatocyte-specific CAND1 deficiency exacerbates HFD-induced hepatic steatosis in female mice. (a) Food intake of CAND1 cKO mice and WT female mice after 12 weeks of NC or HFD feeding (n=7). (b) Body weight in indicated groups (n=7). (c) LW/BW ratio (n=7). (d, e) TG and TC content in indicated groups (n=6). (f) H&E and Oil red O staining of liver sections (n=6). Scale bar=50 µm. Magnification 200×. *p* values obtained two-sided unpaired Student's *t*-test. The data were shown as means ± SD of independent biological replicates. Source data are provided as a Source data file. n.s., not significant difference. LW/BW, liver weight/body weight. TG, Triglyceride. TC, Total cholesterol.

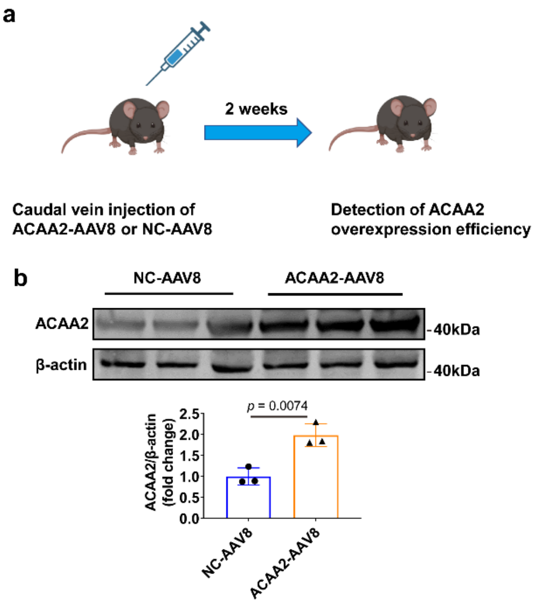

Supplementary Figure 8. Hepatocyte-specific ACAA2 overexpression by ACAA2-AAV8 virus injection. (a) A simple schematic diagram of liver-specific ACAA2 overexpression by injection of ACAA2-AAV8 virus to mice. The diagram was created using BioRender. (b) Western blotting of ACAA2 in NC-AAV8 and ACAA2-AAV8 mice (n=3). *p* values obtained via two-tailed unpaired Student's *t* tests Student's *t*-test. The data were shown as means ± SD of independent biological replicates. Source data are provided as a Source data file.

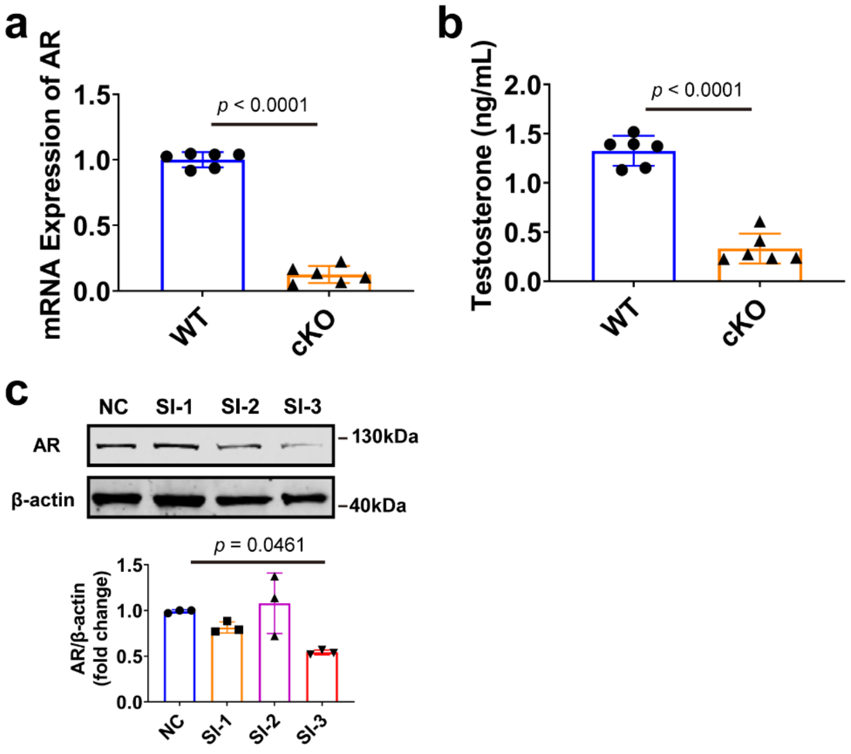

Supplementary Figure 9. The levels of testosterone and AR in cKO mice. (a) mRNA level of AR of WT and cKO mice (n=6). (b) Plasma testosterone levels of WT and cKO mice (n=6). (c) AR knockdown efficiency by Western blotting in AML12 cell (n=3). *p* values obtained via two-tailed unpaired Student's *t* tests, one-way ANOVA with Tukey's multiple comparisons test. The data were shown as means ± SD of independent biological replicates. Source data are provided as a Source data file.

Supplementary Table 1. Binding sites of AR to the promoter region of *CAND1* predicted by JASPAR.

Model ID	Model name	Score	Relative score	Start	End	Strand	predicted site sequence
MA0007.3	Ar	-0.609	0.724156476460345	1797	1813	-1	aaaaaggggctgttctc
MA0007.3	Ar	-0.858	0.721356971058405	2300	2316	1	gagaaagcactctactg
MA0007.3	Ar	1.671	0.749790501827507	2353	2369	-1	cagaaaactttgtaact
MA0007.3	Ar	1.545	0.748373884636163	2353	2369	1	agttacaaagttttctg
MA0007.3	Ar	-0.909	0.720783578385719	2456	2472	1	tggcaccctatgcacac

Supplementary Table 2. Clinical character of participants with normal and NAFLD patients.

	Normal	NAFLD	*P* value
Sex	Male	Male	
Age (years)	39.6 ± 11.89	38 ± 9.81	NS
BMI (kg/m^2)	23.98 ± 1.44	27.7 ± 2.03	0.0033
ALT (U/L)	32 ± 10.95	106.42 ± 33.49	0.0003
AST (U/L)	24.5 ± 5.68	81.85 ± 18.51	< 0.0001
TC (mmol/L)	4.86 ± 0.66	1.93 ± 0.41	< 0.0001
TG (mmol/L)	1.03 ± 0.21	0.97 ± 0.27	NS
Glucose (mmol/L)	4.61 ± 0.51	8.15 ± 0.89	< 0.0001

Clinical and biochemical characteristics of NAFLD and Normal. Data are expressed as mean ± SD. Differences were analyzed by two-tailed unpaired Student's *t* tests. BMI: body mass index; ALT: alanine transaminase; AST: aspartate transaminase; TC: total cholesterol; TG: triglyceride.

Supplementary Table 3. Sequences of Primers used for qRT-PCR.

Company	Primers	Sequences
Seven Biotech	CAND1 (human)	Forward primer: 5'-GACCGTCTCTCCTGCACTAA-3'<br>Reverse primer: 5'-TGGGAACCTGACTCTGAAGC-3'
Seven Biotech	CAND1 (mouse)	Forward primer: 5'-GCGACAAGGACTTCAGGTTC-3'<br>Reverse primer: 5'-GCCAAGGCATTTGACAGCTA-3'
Seven Biotech	GAPDH (human)	Forward primer: 5'-CATGTTCGTCATGGGTGTGAA-3'<br>Reverse primer: 5'-GGCATGGACTGTGGTCATGAG-3'
Seven Biotech	TNF-α (mouse)	Forward primer: 5'-CTGAACTTCGGGGTGATCGG-3'<br>Reverse primer: 5'-GGCTTGTCACTCGAATTTTGAGA-3'
Seven Biotech	IL-6 (mouse)	Forward primer: 5'-CTGCAAGAGACTTCCATCCAG-3'<br>Reverse primer: 5'-AGTGGTATAGACAGGTCTGTTGG-3'
Seven Biotech	IL-1β (mouse)	Forward primer: 5'-GAAATGCCACCTTTTGACAGTG-3'<br>Reverse primer: 5'-TGGATGCTCTCATCAGGACAG-3'
Seven Biotech	ICAM-1 (mouse)	Forward primer: 5'-TGCCTCTGAAGCTCGGATATAC-3'<br>Reverse primer: 5'-TCTGTCGAACTCCTCAGTCAC-3'
Seven Biotech	TLR4 (mouse)	Forward primer: 5'-ATGGCATGGCTTACACCACC-3'<br>Reverse primer:5'-GAGGCCAATTTTGTCTCCACA-3'
Seven Biotech	AR (human)	Forward primer: 5'-CCAGGGACCATGTTTTGCC-3'<br>Reverse primer:5'-CGAAGACGACAAGATGGACAA-3'
Seven Biotech	AR (mouse)	Forward primer: 5'-TCCAAGACCTATCGAGGAGCG-3'<br>Reverse primer:5'-GTGGGCTTGAGGAGAACCAT-3'

Seven Biotech	β-actin (mouse)	Forward primer: 5’-GGCTGTATTCCCCTCCATCG-3’<br>Reverse primer: 5’-CCAGTTGGTAACAATGCCATGT-3’

Supplementary Table 4. Antibodies used for Western bolt.

Antibodies	Dilution ratio	Company	Lot	Clone no.
CAND1	WB 1:1000	Abcam	Ab181216	EPR14242
FBXO42 (IP)	10μg/ml	Santa Cruz	Sc-100737	Monoclonal
β-actin	WB 1:1000	Cell Signaling	#4970	13E5
ACAA2 (IP)	10μg/ml	Santa Cruz	Sc-100847	Monoclonal
FBXO42	WB 1:1000	Abclonal	A14898	Polyclonal
Cullin1	WB 1:1000	Proteintech	12895-1-AP	Polyclonal
Cullin1 (IP)	10μg/ml	Proteintech	12895-1-AP	Polyclonal
ACAA2	WB 1:1000	Abclonal	A15778	A15778
Ubiquitin	WB 1:1000	Santa Cruz	Sc-8017	Monoclonal
