# Supplementary material for: Cullin-associated and neddylation-dissociated protein 1 (CAND1) alleviates NAFLD by reducing ubiquitinated degradation of ACAA2
Source: Nat Commun. 2023 Aug 1;14:4620. doi: 10.1038/s41467-023-40327-5 (PMC10394019; doi:10.1038/s41467-023-40327-5)
Supplement: Supplementary file 3 — Reporting Summary [file 41467_2023_40327_MOESM3_ESM.pdf]

nature portfolio

Corresponding author(s): Zhenwei Pan, Baofeng Yang, Xiangyu Zhong, Yanjie Lu

Last updated by author(s): Jul 11, 2023

Reporting Summary

Nature Portfolio wishes to improve the reproducibility of the work that we publish. This form provides structure for consistency and transparency in reporting. For further information on Nature Portfolio policies, see our Editorial Policies and the Editorial Policy Checklist.

Statistics

For all statistical analyses, confirm that the following items are present in the figure legend, table legend, main text, or Methods section.

n/a	Confirmed	
☐	☒	The exact sample size (*n*) for each experimental group/condition, given as a discrete number and unit of measurement
☐	☒	A statement on whether measurements were taken from distinct samples or whether the same sample was measured repeatedly
☐	☒	The statistical test(s) used AND whether they are one- or two-sided<br>*Only common tests should be described solely by name; describe more complex techniques in the Methods section.*
☒	☐	A description of all covariates tested
☐	☒	A description of any assumptions or corrections, such as tests of normality and adjustment for multiple comparisons
☐	☒	A full description of the statistical parameters including central tendency (e.g. means) or other basic estimates (e.g. regression coefficient) AND variation (e.g. standard deviation) or associated estimates of uncertainty (e.g. confidence intervals)
☐	☒	For null hypothesis testing, the test statistic (e.g. *F*, *t*, *r*) with confidence intervals, effect sizes, degrees of freedom and *P* value noted<br>*Give P values as exact values whenever suitable.*
☒	☐	For Bayesian analysis, information on the choice of priors and Markov chain Monte Carlo settings
☒	☐	For hierarchical and complex designs, identification of the appropriate level for tests and full reporting of outcomes
☒	☐	Estimates of effect sizes (e.g. Cohen's *d*, Pearson's *r*), indicating how they were calculated

Our web collection on statistics for biologists contains articles on many of the points above.

Software and code

Policy information about availability of computer code

Data collection	No software was used.
Data analysis	R 4.1.2; Graphpad Prism 8.2.0

For manuscripts utilizing custom algorithms or software that are central to the research but not yet described in published literature, software must be made available to editors and reviewers. We strongly encourage code deposition in a community repository (e.g. GitHub). See the Nature Portfolio guidelines for submitting code & software for further information.

Data

Policy information about availability of data

All manuscripts must include a data availability statement. This statement should provide the following information, where applicable:

- Accession codes, unique identifiers, or web links for publicly available datasets
- A description of any restrictions on data availability
- For clinical datasets or third party data, please ensure that the statement adheres to our policy

The mass spectrometry proteomics data have been deposited to the ProteomeXchange Consortium via the PRIDE partner repository with the dataset identifier PXD043719. The previously published data sets re-analysed in this study were obtained from Gene Expression Omnibus (GEO), through the accession code (GSE126848). All other data generated or analyzed in this study are available within the article and its supplementary information files. Source data are provided with this paper.

Research involving human participants, their data, or biological material

Policy information about studies with human participants or human data. See also policy information about sex, gender (identity/presentation), and sexual orientation and race, ethnicity and racism.

Reporting on sex and gender	For the detection of CAND1 in liver samples (Figure 1A, B), male patients were considered in this study. This study involves 6 liver samples of male normal donors and 7 liver samples of male NAFLD patients.<br>For the analysis of GEO database (GSE126848, Supplementary Figure 1), data of 14 male normal, and 12 male NASH and 9 male NAFLD patients were involved in this analysis
Reporting on race, ethnicity, or other socially relevant groupings	For the detection of CAND1 in liver samples (Figure 1A, B), male patients were recruited by Second Affiliated Hospital of Harbin Medical University (Harbin, China), there is no potential bias for recruiting patient cohort.
Population characteristics	Clinical character of participants with normal and NAFLD patients

	Normal	NAFLD
Sex	Male	Male
Age (years)	39.6 ± 11.89	38 ± 9.81
BMI (kg/m2)	23.98 ± 1.44	27.7 ± 2.03
ALT (U/L)	32 ± 10.95	106.42 ± 33.49
AST (U/L)	24.5 ± 5.68	81.85 ± 18.51
TC (mmol/L)	4.86 ± 0.66	1.93 ± 0.41
TG (mmol/L)	1.03 ± 0.21	0.97 ± 0.27
Glucose (mmol/L)	4.61 ± 0.51	8.15 ± 0.89

Recruitment	Patients were recruited by Second Affiliated Hospital of Harbin Medical University (Harbin, China). No expect biases, including self-selection bias was expected from patients samples. Informed written consents were obtained from all participants and all investigations conformed to the principles of the Declaration of Helsinki. The study protocols were procured in accordance with the guidelines of and approved by the Ethics Committee of the Harbin Medical University.
Ethics oversight	The experimental protocols involving the human sample in this study were approved by the Research Ethics Committee of the institutional Ethics Committees of Harbin Medical University (HMUIRB5025722).

Note that full information on the approval of the study protocol must also be provided in the manuscript.

Field-specific reporting

Please select the one below that is the best fit for your research. If you are not sure, read the appropriate sections before making your selection.

☒ Life sciences ☐ Behavioural & social sciences ☐ Ecological, evolutionary & environmental sciences

For a reference copy of the document with all sections, see nature.com/documents/nr-reporting-summary-flat.pdf

Life sciences study design

All studies must disclose on these points even when the disclosure is negative.

Sample size	No statistical test was used to predetermine sample size. Sample size was determined based on the experimental results that we obtained from preliminary experiments. In vivo studies, we used standard sample sizes reported in the literature previously in mouse studies (Reference: PMID: 36918564; 29291351; 29227477; 33397952; 34893641). The numbers of performed experiments were indicated in each figure legend. The sample size for whole animal experiments was set to be >3 mice for each group, and for molecular biology experiments.
Data exclusions	The data from the animals died before the completion of the whole experimental procedures were excluded from our data analysis.
Replication	For cellular and molecular experiments, each single measurement was performed in triplicate and the results were consistently reproducible.
Randomization	All animals and cells were randomly assigned to experimental groups.
Blinding	The experimental designers and experimenters/data analysts were double blinded.

Reporting for specific materials, systems and methods

We require information from authors about some types of materials, experimental systems and methods used in many studies. Here, indicate whether each material, system or method listed is relevant to your study. If you are not sure if a list item applies to your research, read the appropriate section before selecting a response.

Materials & experimental systems

n/a	Involved in the study
	[x] Antibodies
	[x] Eukaryotic cell lines
[x]	Palaeontology and archaeology
	[x] Animals and other organisms
[x]	Clinical data
[x]	Dual use research of concern
[x]	Plants

Methods

n/a	Involved in the study
[x]	ChIP-seq
[x]	Flow cytometry
[x]	MRI-based neuroimaging

Antibodies

Antibodies used	CANDI. (WB, 1:1000, Abcam, Ab181216); FBXO42 (IP, 10 g/ml, Santa Cruz, Sc-100737); β-actin (WB, 1:1000, Cell Signaling, #4970) ACAA2 (IP, 10 g/ml, Santa Cruz, Sc-100847); FBXO42 (WB, 1:1000, Abclonal, A14898); Cullin1 (WB, 1:1000, Proteintech, 12895-1-AP); Cullinl (IP, 10 g/ml, Proteintech, 12895-1-AP); ACAA2 (WB, 1:1000, Abclonal, A15778); Ubiquitin (WB, 1:1000, Santa Cruz, Sc-8017).
Validation	All the primary antibody for the species and application statement on the manufacturer's websites.<br>Recombinant Anti-CAND1 antibody [EPR14242(B)] - C-terminal (ab181216)<br>Validated in our paper published before.<br>https://www.abcam.com/products/primary-antibodies/cand1-antibody-epr14242b-c-terminal-ab181216.html<br>Applications: WB, IHC-P, ICC/IF, IP, Flow Cyt (Intra)<br>Reactivity: Human.<br>FBXO42 Antibody (FL-6): sc-100737<br>Validated by other users, cited 4 times in the company website.<br>https://www.scbt.com/p/fbxo42-antibody-fl-6<br>Applications: WB, IP and ELISA.<br>Reactivity: mouse, rat and human.<br>β-Actin (13E5) Rabbit mAb #4970<br>Validated by other users, cited 6477 times in the company website.<br>https://www.cellsignal.com/products/primary-antibodies/b-actin-13e5-rabbit-mab/4970<br>Applications: WB-Western Blot IP-Immunoprecipitation IHC-Immunohistochemistry ChIP-Chromatin Immunoprecipitation C&R-CUT&RUN C&T-CUT&Tag DB-Dot Blot eCLIP-eCLIP IF-Immunofluorescence F-Flow Cytometry.<br>Reactivity: All Species Expected.<br>ACAA2 Antibody (192): sc-100847<br>Validated by other users, cited 7 times in the company website.<br>https://www.scbt.com/p/acaa2-antibody-192?requestFrom=search<br>Applications: WB, IP, IF, IHC(P) and ELISA.<br>Reactivity: mouse, rat and human.<br>FBXO42 Rabbit pAb (A14898)<br>Validated in our paper published before.<br>https://abclonal.com/catalog-antibodies/FBXO42RabbitpAb/A14898<br>Applications: WB.<br>Reactivity: mouse.<br>CUL1 Polyclonal antibody: 12895-1-AP<br>Validated by other users, cited 13 times in the company website.<br>https://www.ptgcn.com/products/CUL1-Antibody-12895-1-AP.htm<br>Applications: FC, IHC, IP, WB, ELISA.<br>Reactivity: Human, Mouse.<br>ACAA2 Rabbit pAb (A15778)<br>Validated by other users, cited 1 times in the company website.<br>https://abclonal.com/catalog-antibodies/ACAA2RabbitpAb/A15778<br>Applications: WB, IHC-P, IF/ICC.<br>Reactivity: Human, Mouse, Rat.<br>Ubiquitin Antibody (P4D1): sc-8017<br>Validated by other users, cited 3193 times in the company website.<br>https://www.scbt.com/p/ubiquitin-antibody-p4d1?requestFrom=search<br>Applications: WB, IP, IF, IHC(P), FCM and ELISA.<br>Reactivity: mouse, rat, human and Drosophila.

Eukaryotic cell lines

Policy information about cell lines and Sex and Gender in Research

Cell line source(s)	AML12 and HepG2 cells were purchased from Procell Life Science&Technology Co., Ltd. THLE-2 cell was purchased from iCell Bioscience Inc.
Authentication	STR profiling is used to authenticate these cell lines.
Mycoplasma contamination	All cell lines and organoid lines were negative for mycoplasma contamination and routinely tested for mycoplasma using PCR assay.
Commonly misidentified lines (See ICLAC register)	None of the cell lines used were misidentified.

Animals and other research organisms

Policy information about studies involving animals; ARRIVE guidelines recommended for reporting animal research, and Sex and Gender in Research

Laboratory animals	6-8 weeks old mice were used for experiments. Global CAND1 knockout mice, CAND1 flox/flox (CAND1-flox) mice, CAND1 conditional knockin mice, Alb-cre mice were generated by Cyagen Biosciences Inc (Guangzhou, China). All animals were maintained in accordance with guidelines: Standard chow diet and water were offered ad libitum; for housing sterilized plastic cages under specific pathogen-free conditions were used; as housing conditions 22 ± 2 °C, 12/12 light/dark cycle, 55 ± 10% humidity and <400 lux was maintained.
Wild animals	The study does not involve any wild animals
Reporting on sex	NAFLD is a sexual dimorphic disease and the prevalence and severity of NAFLD are affected by sex. In adult populations, NAFLD prevalence is higher in men than in premenopausal women. Therefore, only NAFLD male patients were selected for analysis in GEO data(GSE126848, Supplementary Figure 1) and detection of CAND1 in liver samples (Figure 1A, B). However, the regulation of CANDI. on NAFLD is not sexual dimorphic, as deletion of CAND1 also exacerbated NAFLD development in female mice.
Field-collected samples	The study does not involve any samples collected from Field.
Ethics oversight	The experimental protocols involving the use of animals in this study were approved by the Animal Care and Use Committee of Harbin Medical University (HMUIRB5025722)

Note that full information on the approval of the study protocol must also be provided in the manuscript.
